# Activate to Eradicate: Inhibition of *Clostridium difficile* Spore Outgrowth by the Synergistic Effects of Osmotic Activation and Nisin

**DOI:** 10.1371/journal.pone.0054740

**Published:** 2013-01-22

**Authors:** Michelle M. Nerandzic, Curtis J. Donskey

**Affiliations:** 1 Research Service, Cleveland Veterans Affairs Medical Center, Cleveland, Ohio, United States of America; 2 Geriatric Research, Education and Clinical Center, Cleveland Veterans Affairs Medical Center, Cleveland, Ohio, United States of America; University of Connecticut, United States of America

## Abstract

**Background:**

Germination is the irreversible loss of spore-specific properties prior to outgrowth. Because germinating spores become more susceptible to killing by stressors, induction of germination has been proposed as a spore control strategy. However, this strategy is limited by superdormant spores that remain unaffected by germinants. Harsh chemicals and heat activation are effective for stimulating germination of superdormant spores but are impractical for use in a hospital setting, where *Clostridium difficile* spores present a challenge. Here, we tested whether osmotic activation solutes will provide a mild alternative for stimulation of superdormant *C. difficile* spores in the presence of germinants as previously demonstrated in several species of *Bacillus*. In addition, we tested the hypothesis that the limitations of superdormancy can be circumvented with a combined approach using nisin, a FDA-approved safe bacteriocin, to inhibit outgrowth of germinated spores and osmotic activation solutes to enhance outgrowth inhibition by stimulating superdormant spores.

**Principal Findings:**

Exposure to germination solution triggered ∼1 log_10_ colony forming units (CFU) of spores to germinate, and heat activation increased the spores that germinated to >2.5 log_10_CFU. Germinating spores, in contrast to dormant spores, became susceptible to inhibition by nisin. The presence of osmotic activation solutes did not stimulate germination of superdormant *C. difficile* spores exposed to germination solution. But, in the absence of germination solution, osmotic activation solutes enhanced nisin inhibition of superdormant spores to >3.5 log_10_CFU. The synergistic effects of osmotic activation solutes and nisin were associated with loss of membrane integrity.

**Conclusions:**

These findings suggest that the synergistic effects of osmotic activation and nisin bypass the limitations of germination as a spore control strategy, and might be a novel method to safely and effectively reduce the burden of *C.difficile* spores on skin and environmental surfaces.

## Introduction

*Clostridium difficile* is an anaerobic, spore-forming bacterium that is the major cause of healthcare-associated diarrhea, ranging from mild diarrhea to fulminant colitis [1], [2]. While the vegetative form of *C.difficile* is responsible for producing the toxins that cause disease, the spore form is the major source of transmission [3]. Unlike vegetative *C. difficile*, dormant spores are resistant to oxygen, desiccation and many agents commonly employed to eradicate pathogens from skin and environmental surfaces [4], [5]. Therefore, spores may remain on skin and surfaces for extended periods of time, presenting a difficult challenge for infection control programs in hospitals and nursing homes [6], [7]. Spore disinfection techniques are available to address environmental contamination, but each method has disadvantages that range from being time-consuming and expensive to being corrosive to surfaces and having only modest efficacy [8]–[11]. Moreover, there are no established methods for significantly reducing the burden of *C. difficile* spores from patient and healthcare workers’ skin. Given these limitations, there is a need for new strategies to safely and effectively reduce the burden of *C. difficile* spores on skin and surfaces.

Perpetuation of *C. difficile’s* pathogenic cycle is dependent on the dormant spore’s ability to sense its surroundings and germinate when conditions become favorable. The germination process is required to allow outgrowth of vegetative cells and is defined as the irreversible loss of spore-specific properties [12], [13]. As the spore proceeds through germination, it becomes more susceptible to killing by heat and other stressors. Consequently, induction of germination has been proposed as a strategy to facilitate eradication of spores. As early as the 1960s, this strategy was studied in *Bacillus* and *Clostridium* spp. as a possible means to enhance spore killing in food and on surfaces by heat, radiation, and several compounds [14]–[20]. More recently, Lowden et al. coined the phrase “germinate to exterminate” to describe this approach for *C. difficile* and demonstrated that triggering germination in a rich medium containing a reducing agent (thioglycollate) facilitated killing by desiccation and aerobic conditions (European Society of Clinical Microbiology and Infectious Diseases, Helsinki, Finland, 2009, Abstract number: P1260). We subsequently demonstrated that triggering germination in solution without a reducing agent enhanced *C. difficile* spore killing by ultraviolet-C (UV-C) and heat [21]. Additionally, we showed reduced survival of the germinated spores on surfaces in room air, possibly due to increased susceptibility to stressors such as oxygen and desiccation [21]. Compounds such as nisin have also been studied for synergistic effects on killing of germinated spores [22], [23]. Nisin is an FDA-approved, GRAS (“generally regarded as safe”) bacteriocin that is commonly used as a food preservative [24]. Nisin has previously been reported to prevent outgrowth of germinating spores produced by several species of *Clostridium* and *Bacillus*, including *B. anthracis* [25], [26]. Because it is regarded as safe, nisin has the potential to be utilized on skin and environmental surfaces; however, it is not known if nisin is similarly effective for outgrowth inhibition of germinated *C. difficile* spores.

Despite the promise of the “germinate to exterminate” strategy, it is met with distinct challenges. One major challenge is that in order for this strategy to be efficacious, spores must be exposed to the appropriate germinant triggers. Our current understanding of spore germination is based primarily on *Bacillus spp.*, which has been studied in great detail. However, much less is known about germination in *Clostridium spp.* and in particular *C. difficile*. Moreover, there is ambiguity in the literature regarding the defined elements that are effective for stimulating *C. difficile* germination. While glycine and taurocholic acid are effective co-germinants for some strains, other studies found that several amino acids stimulate *C. difficile* germination [27], [28]. Recently, it has been suggested that multiple strains should be examined due to *C. difficile’s* diversity in response to germinants [29]. Therefore, when germination of unknown strains of *C. difficile* spores is requisite (i.e. spore contamination in the hospital), the germinant triggers must be comprehensive and induce germination across diverse strains of *C.difficile*.

Another considerable challenge is the heterogeneous dormancy of spore populations (i.e. superdormant spores) [12]. Although we previously demonstrated that germination enhanced killing of *C. difficile* spores by some stressors, a superdormant fraction of spores remained that was unaltered by exposure to germinants [21]. While all spores are initially dormant, some spores respond to germinants more readily than others [12], [30]. Experiments designed to completely germinate populations of spores (via germinant receptors) reveal that a portion of spores consistently remains which does not germinate [31]. Nonetheless, there are procedures for bypassing this limitation. Heat activation of spores (60–115°C, ranging from minutes to several hours) is commonly used in germination experiments to stimulate and synchronize the germination response [32]. Enzymatic treatments or compounds such as dodecylamine are also effective for circumventing the germination machinery and stimulating superdormant spores to germinate [12], [33]. However, the severity of these procedures prevents them from being safe or practical for use on skin and environmental surfaces in the hospital.

One potential alternative strategy for stimulating dormant spores to germinate is osmotic activation. Solutes which osmotically permeate plasma membranes (non-polar, small, hydrophobic) have been demonstrated to effectively stimulate germination of spores in several species of *Bacillus* [34]. Mechanistically, as demonstrated in *Bacillus cereus*, osmotic activation occurs when uncharged forms of relevant solutes permeate the spore core and react inside, thereby raising the internal osmotic pressure [35]. Similarly, heat activation is also achieved by increasing internal osmotic pressure (via heat induced core mineral solubility changes) [35]. Ammonium hydroxide and tris(hydroxymethyl)aminomethane hydrochloride (Tris HCl) are solutes that have been shown to successfully stimulate unactivated spores of several *Bacillus spp*. [34], [35]. However, it is unknown if these solutes will be similarly efficacious for stimulating germination of dormant *C. difficile* spores.

Here, we suggest a safe and practical means to overcome some of the limitations of the “germinate to exterminate” strategy. By applying benign compounds to stimulate germination of dormant and superdormant spore fractions, we postulate that outgrowth inhibition will be enhanced when exposed to nisin, providing a safe and effective means to reduce spores on skin and environmental surfaces. Accordingly, to address diversity in germinants among strains of *C. difficile*, we initially investigated the germination response in a broad collection of *C. difficile* strains when spores were exposed to a comprehensive germination solution (Table 1) under ambient conditions (room-air, 22°C). Next, we tested the hypothesis that nisin will inhibit outgrowth of *C. difficile* spores triggered to germinate in room air. Finally, to address difficult to germinate fractions of spores, we assessed osmotic activation as a means to stimulate germination of superdormant spores and elicit enhanced inhibition of *C. difficile* spores by nisin. Additionally, we propose a potential mechanism by which osmotic activation is effective for nisin-induced inhibition of *C. difficile* spore outgrowth.

**Table 1:** Formulation of *Clostridium difficile* spore germination solution consisting of amino acids, minerals and taurocholic acid prepared in sterile deionized water.

Component	Concentration (mg/L)	Component	Concentration (mg/L)
Amino Acid		Mineral	
Histidine	100	KH_2_PO_4_	300
Trytophan	100	Na_2_HPO_4_	1500
Glycine	100	NaCl	90
Tyrosine	100	CaCl_2_.2H_2_O	26
Arginine	200	MgCl_2_.6H_2_O	20
Phenylalanine	200	MnCl_2_.4H_2_O	10
Methionine	200	(NH_4_)_2_SO_4_	40
Threonine	200	FeSO_4_.7H_2_O	4
Alanine	200	CoCl_2_.6H_2_O	1
Lysine	300	NaHCO_3_	5000
Serine	300		
Valine	300	**Bile Salt**	
Isoleucine	300	Taurocholic acid	1000
Aspartic acid	300		
Leucine	400		
Cysteine	500		
Proline	600		
Glutamic acid	900		

## Materials and Methods

### Ethics Statement

Bacterial strains were isolated from patients at the Cleveland VA Medical Center. The Institutional Review Board of the Cleveland VA Medical Center approved the study protocol for collection of all patient isolates. Informed consent was not obtained because the isolates were cultured from clinical samples with no collection of patient identifiers or interaction with subjects.

### *C. difficile* Strains

Eleven *C. difficile* strains from the American Type Culture Collection (ATCC) and 2 strains cultured from patients with CDI in Cleveland were studied. ATCC 43593, 43601, 43603, and 700057 are non-toxigenic strains (tcdA−, tcdB−) from ribotypes 060, 031, 085, and 038, respectively. ATCC 43594, 43596, 43597, 43599, 43600 and BBA-1382 (630) are toxigenic (tcdA+, tcdB+), non-epidemic (cdtB−) strains from ribotypes 005, 012, 014, 001, 014 and 012, respectively. ATCC 43598 is a non-epidemic (cdtB−), toxigenic variant that is tcdB+ and tcdA− from ribotype 017. The two clinical isolates are toxigenic (tcdA+, tcdB+) strains. VA 17 is an epidemic (cdtB+) restriction endonuclease analysis (REA) BI strain and VA 11 is a non-epidemic (cdtB−) REA J strain.

### Preparation of *C. difficile* Spores

It is our experience that *C. difficile* spore preparations stored at or below 4°C release and accumulate Ca(2+)-dipicolinic acid, which causes changes in the spore’s dormancy and germination response over time (authors’ unpublished data). Therefore, to eliminate heterogeneity in germination response imposed by preparation and storage methodology, all spores were prepared and stored under the conditions described below.

Sporulation was induced in a Whitley Workstation MG1000 anaerobic chamber (Microbiology International, Frederick, MD) at 37°C with 75% humidity as previously described by Sorg et al. [3], with the following modifications. *C. difficile* spores were streaked for isolation on a pre-reduced *Clostridium difficile* Brucella agar (CDBA) selective plate [36] and incubated for 24 hours. Isolated colonies were suspended into pre-reduced phosphate buffered saline (PBS) to a 0.8 McFarland. Pre-reduced Brain-heart infusion (BHIS) plates were spread with 100 µl of the *C. difficile* suspension and incubated for one week. Spores were harvested from the plates using sterile swabs and 8 mL of ice-cold, sterile, distilled water. Spores were washed five times by centrifuging at 15,000×g for 5 min and re-suspending in 10 mL of ice-cold, sterile, distilled water. After washing, the spores were collected in 1 mL of 20% (wt/vol) HistoDenz (Sigma, St. Louis, MO) and layered onto 20 mL of 50% (wt/vol) HistoDenz solution. The gradient was centrifuged at 15,000×g for 15 min and the spore pellet was carefully collected from the bottom of the tube. Spores were washed three times as described above and aliquots were stored at −80°C in sterile water until use. Each aliquot was frozen once after harvesting to avoid freeze-thaw damage. Aliquots were thawed on ice and used immediately for each experiment. Prior to testing, spore preps were confirmed by phase contrast microscopy and malachite green staining to be >99% dormant, bright-phase spores.

### Examination of Germination in Unactivated and Heat Activated *C. difficile* Spores

Previous data suggests that defined germinants may vary among strains or even isolates of *C. difficile* [29]. It is also our experience that spore strains show a great deal of variety in their response to germinants such as amino acids, minerals, and taurocholic acid (authors’ unpublished data). Therefore, to elicit a similar germination response among multiple strains, a comprehensive medium consisting of 18 L-amino acids, 10 minerals and taurocholic acid was used to induce germination (Table 1).

We assessed the germination response of heat activated spores versus spores in their natural state (unactivated). For germination experiments, 10^6^ CFU of spores (ten µL) were incubated in one mL of germination solution under ambient conditions for 30 minutes (room air, approximately 22°C). Thirteen strains of *C. difficile* spores were tested (described above in *C. difficile strains*). Each strain of spore was split into two sets. One set of spores was added directly to the germination medium without heat activating. The other set was heat activated at 80°C for ten minutes and then added to the germination medium. Germination was confirmed by three methods: 1) heat susceptibility at 80°C for 5 minutes 2) bright (dormant spores) to dark (germinating spores) phase transition under phase contrast microscopy and 3) a modified Wirtz-Conklin stain was performed as previously described by Hamouda et al. [37] to differentiate between dormant (green) or germinated (pink) spores. Experiments were performed in triplicate.

### Inhibition of Germinated *C. difficile* Spores by Nisin

Vegetative *C. difficile* is acutely sensitive to the activity of nisin [38]. Therefore, to examine the effects of nisin on the inhibition of spore outgrowth without introducing error due to vegetative inhibition, initial experiments were conducted to determine the minimum inhibitory concentrations (MICs) for the thirteen *C. difficile* strains examined. The MICs were found to be consistent for all strains tested (MIC_90_ of 0.3 µg/mL). As a result, the working concentration of nisin (MP Biomedicals, Solon, Ohio) utilized for the following experiments was 3.2 µg/mL (stock contained 2.5% w/w nisin, 1000 U/mg). This concentration was found to be sufficient for outgrowth inhibition of 10^6^ CFU of spores while being low enough to yield vegetative growth after diluting and plating for colony counts (data not shown).

Because all 13 *C. difficile* isolates had equivalent MICs to nisin and similar germination responses to the medium described above, VA 11, VA 17, ATCC 700057 and BBA-1382 were chosen as representative strains for this experiment. Each strain was split into two sets; one set was heat-activated at 80°C for 10 minutes and the other was not. Ten µL (10^6^ CFU) of unactivated or heat activated spores were immersed in either one mL water (positive control), nisin (3.2 µg/mL) suspended in water, germination medium (Table 1), or germination medium containing nisin (3.2 µg/mL). After 30 minutes of incubation under ambient conditions (room air, 22°C), aliquots of each spore suspension were either transferred directly to an anaerobic chamber or heat shocked (80°C for 5 minutes) prior to transfer to assess heat susceptibility. Samples were serially diluted and plated on CDBA. Following 48 hours of incubation, colonies of *C. difficile* were counted and the log_10_CFU of spores recovered from solution compared to baseline (water control) was recorded.

Germination was confirmed by two methods: 1) bright (dormant spores) to dark (germinating spores) phase transition under phase contrast microscopy and 2) quantifying dipicolinic acid (DPA) release in a fluorescence plate reader by measuring the formation of Tb^3+^-DPA as previously described by Ammann et al [39]. In brief, DPA release was measured by mixing 100 *µ*l of spore suspension supernatants (after centrifugation) with 100 *µ*l terbium chloride solution (TbCl_3_, 30 *µ*M) in a black 96-well microtiter plate (in 8 replicates). Fluorescence was immediately measured and recorded using a plate reader (SpectraMaxM2, Bucher Biotec, Basel, Switzerland) at an excitation wavelength of 272 nm and emission wavelength of 545 nm. Germination was indicated by increase in relative fluorescence units (RFU) in spore supernatants for each solution compared to spore supernatant for water (negative control). The experiments were repeated three times.

### Synergistic Effects of Nisin and Osmotically Activated *C. difficile* Spores

We assessed osmotic activation solutes for stimulation of unactivated *C.difficile* spores. Experiments were performed as described in *Inhibition of Germinated C. difficile Spores by Nisin* with the following modifications. The osmotic activation solutes assessed were ammonium hydroxide (7.1 mM), tris(hydroxymethyl)aminomethane hydrochloride (Tris HCl (1.5M)), and glycerol (1.5 M). Sucrose (1.5 M), a solute which does not readily permeate plasma membranes and osmotically dehydrates the spore core, served as a negative control [35]. Spores were incubated in either water, solute, germination medium containing a solute, or nisin (3.2 µg/mL) containing a solute.

### Evidence for Membrane Integrity as Mechanism of Inhibition by Nisin and Osmotic Activation

Disruption of membrane integrity was evaluated by incubating spores in the presence of 0.6 µM propidium iodide (PI; Invitrogen, Grand Island, New York) and monitoring increases in membrane permeability by measuring the up-take of PI by flow cytometry as previously described by Gut et al. with the following modifications [20]. Ten µL (10^6^ CFU) of unactivated spores (VA 11, VA 17, ATCC 700057 and BBA-1382) were immersed in either one mL water, germination medium (Table 1), germination medium with nisin (3.2 µg/mL), germination medium with nisin and solute, solute and nisin, or solute alone. Spores incubated in water without PI served as baseline. The solutes examined were compounds that either readily permeate plasma membranes (7.1 mM ammonium hydroxide, 1.5 M Tris HCl and 1.5 M glycerol) or that do not permeate plasma membranes (1.5 M sucrose and 0.9 M sodium chloride (NaCl). After 30 minutes of incubation, the *C. difficile*-associated PI fluorescence was measured by a BD LSR II flow cytometer (BD Biosciences, San Jose, California) with excitation at 488 nm with a Coherent Sapphire blue laser and fluorescence emission through a band-pass filter at 675/20 nm. For each sample, 10,000 events were detected and the data were analyzed using BD FACSDiva 6.0 software (BD Biosciences, San Jose, California). The experiments were performed in triplicate and the data were plotted as percent increase in the geometric mean of the fluorescence intensity (MFI).

### Data Analysis

Data were analyzed using STATA 9.0 (StataCorp, College Station, TX). Continuous data were analyzed using unpaired *t* tests and categorical data were assessed using Fisher’s exact test.

## Results

### Examination of Germination in Unactivated and Heat-activated *C. difficile* Spores

**Figure 1:**
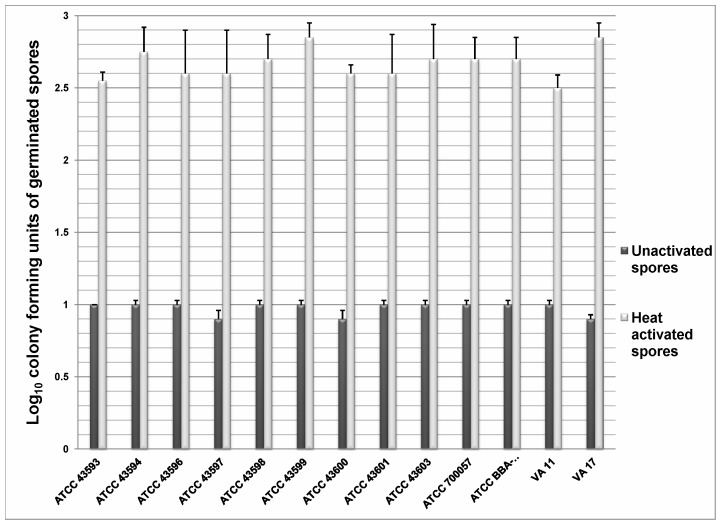
Germination in unactivated and heat activated *C. difficile* spores. The log_10_CFU of unactivated and heat activated spores that germinated in the comprehensive germination medium (Table 1) after 30 minutes of incubation under ambient conditions. Spores were confirmed as dormant or germinated by phase contrast microscopy, heat susceptibility (80°C for 5 minutes) and a modified Wirtz-Conklin stain. The means of the data from experiments conducted in triplicate are presented. Error bars indicate standard error.

Figure 1 shows the log_10_CFU of spores that germinated (heat susceptible, dark phase, stained pink) in the comprehensive germination medium either with or without heat activation. There was no significant difference in the germination response among the 13 strains of unactivated (P>0.1) or heat activated spores tested (P>0.9). Approximately 1 log_10_CFU (range: 0.9 log_10_CFU to 1.1 log_10_CFU) of the unactivated spores germinated in the germination medium, whereas >2.5 log_10_CFU of all 13 strains (range: 2.5 log_10_CFU to 2.9 log_10_CFU) germinated after heat activation (P<0.001 compared to unactivated spores for both comparisons), consistent with germination of the superdormant fraction of spores in response to heat activation.

### Inhibition of Germinated *C. difficile* Spores by Nisin

**Figure 2:**
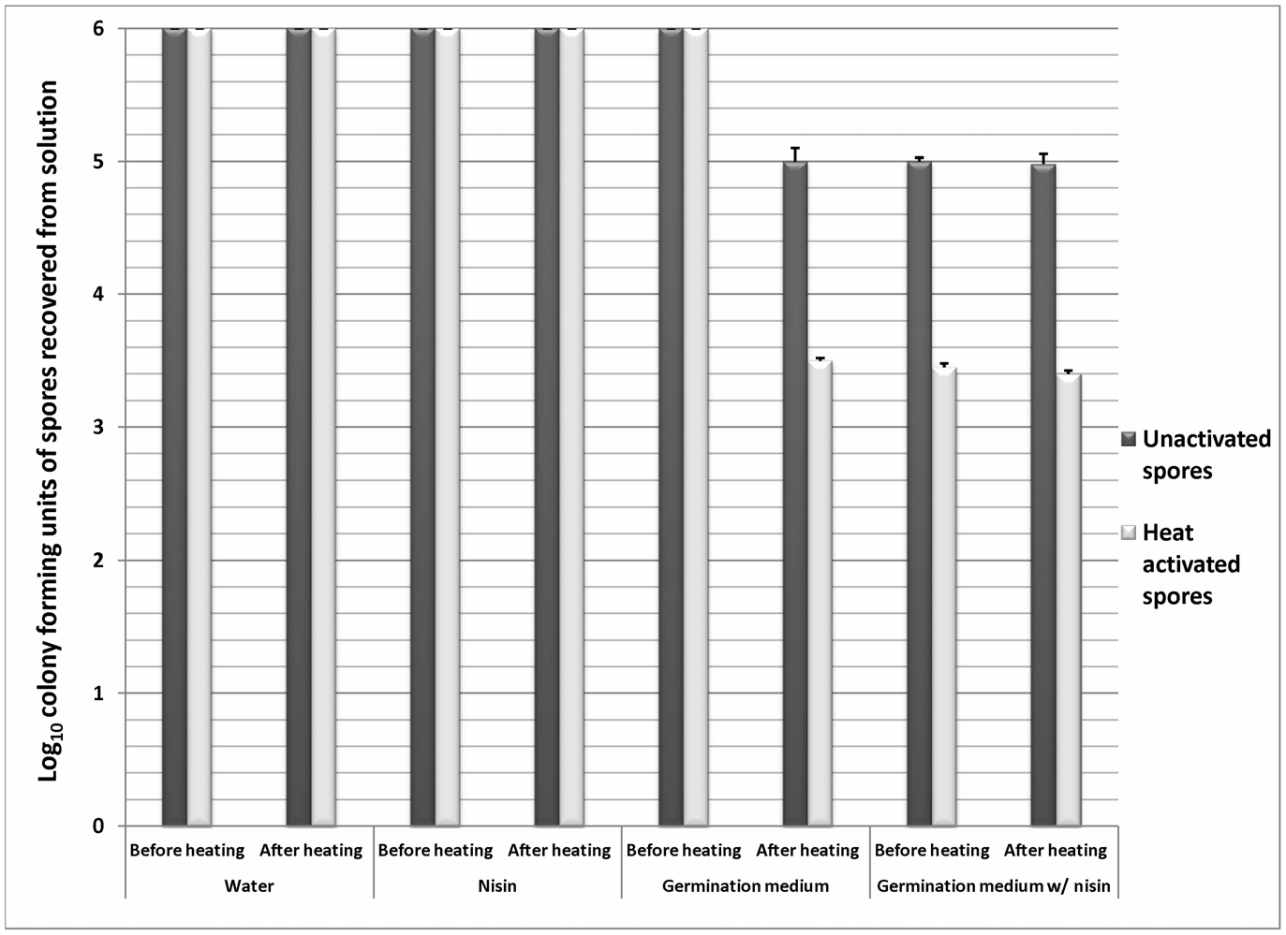
Inhibition of germinated *C. difficile* spores by nisin. The log_10_CFU of unactivated and heat activated spores that were recovered from solutions after 30 minutes of incubation under ambient conditions. Spore suspension were either plated before heating or after heating at 80°C for 5 minutes (kills germinated but not dormant spores). Spores were confirmed as dormant or germinated by phase contrast microscopy and DPA release. The means of the data from experiments conducted in triplicate are presented. Error bars indicate standard error.

Figure 2 shows the log_10_CFU of unactivated and heat activated spores that were recovered from solutions before and after heat treatment (80°C for 5 min kills germinated [dark phase] but not dormant [bright phase] spores). As noted previously, exposure to germination solution triggered ∼1 log_10_CFU of the spores to germinate (dark phase, DPA release) and become susceptible to killing by heat, and heat activation increased the percentage of spores that germinated to >2.5 log_10_CFU (P<0.001 compared to unactivated spores for both comparisons). Nisin alone did not stimulate germination (i.e., spores remained bright phase and heat resistant) or kill spores. However, germinating spores became susceptible to outgrowth inhibition by nisin in room air (i.e., ∼1 log_10_CFU of unactivated spores and >2.5 log_10_CFU of heat activated spores were killed by nisin). Germinating spores exposed to nisin were washed four times with sterile deionized water to confirm that the inhibitory effect remained after removing nisin from the spore suspensions. Consequently, after washing spores free of nisin, the inhibitory effect persisted and dark phase spores were prevented from outgrowing on culture medium (data not shown).

### Synergistic Effects of Nisin and Osmotically Activated *C. difficile* Spores

**Figure 3:**
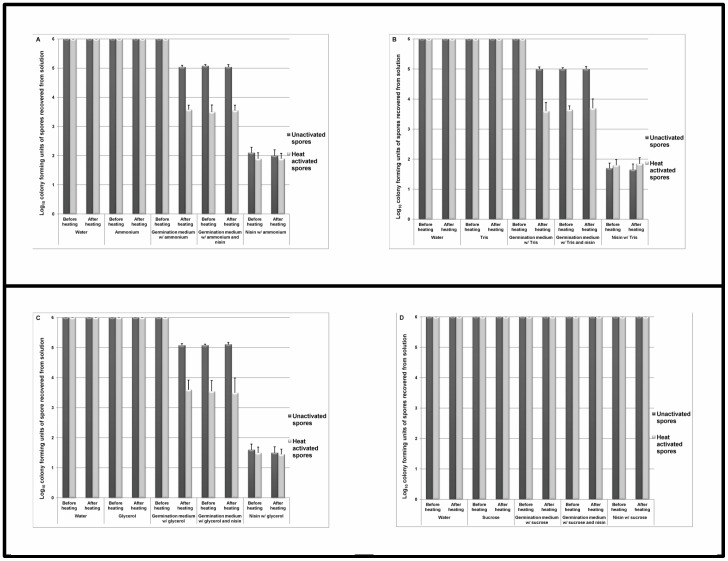
Synergistic effects of nisin and osmotically activated *C. difficile* spores. The log_10_CFU of unactivated and heat activated spores that were recovered from solutions after 30 minutes of incubation under ambient conditions. Spore suspension were either plated before heating or after heating at 80°C for 5 minutes (kills germinated but not dormant spores). Figure 3A–C shows solutions containing solutes that readily permeate plasma membranes (ammonium hydroxide, TRIS HCl, and glycerol). Figure 3D shows solutions containing sucrose which does not permeate plasma membranes. Spores were confirmed as dormant or germinated by phase contrast microscopy and DPA release. The means of the data from experiments conducted in triplicate are presented. Error bars indicate standard error.

Four solutes were assessed for their ability to osmotically activate *C. difficile* spores and augment the inhibitory effects of nisin on germinated spores. The log_10_CFU of unactivated and heat activated spores that were recovered from solutions before and after heat treatment was determined. Figure 3A–C shows the results for solutes that readily permeate plasma membranes (ammonium hydroxide, TRIS HCl, and glycerol). Contrary to previous findings for *Bacillus* spp. [34], the presence of these solutes in a germinant solution (Table 1) did not stimulate germination of unactivated *C. difficile* spores or enhance germination of heat activated spores. Accordingly, there was no increase in the quantity of germinated spores that were inhibited by nisin in room air (∼1 log_10_CFU). Unexpectedly, however, nisin and solute alone significantly increased inhibition of spores in room air (P<0.001 compared to heat activated spores in germination solution containing nisin and solute). While heat activation increased nisin inhibition from ∼1 log_10_CFU to >2.5 log_10_CFU, solute alone in the presence of nisin further enhanced inhibition to >3.5 log_10_CFU (range: 3.6 log_10_CFU to 4.8 log_10_CFU). Interestingly, spores remained dormant (bright phase, heat resistant, no DPA release) in solute alone, whereas in the presence of solute with nisin spores transitioned to dark phase and released DPA (>100-fold increase in RFU). Spores exposed to nisin and solute were washed four times with sterile deionized water to confirm that the inhibitory effect remained after removing nisin and solute from the spore suspensions. Consequently, after washing spores free of nisin and solute the inhibitory effect persisted and dark phase spores were prevented from growing on culture medium (data not shown).

Figure 3D shows the results for sucrose, a solute which does not readily permeate plasma membranes (negative control). Spores remained heat resistant in all sucrose solutions tested. Unlike solutes which readily permeate membranes, sucrose combined with nisin did not induce outgrowth inhibition or dark phase transition of spores (spores remained dormant, no DPA release). In germination solution containing glycerol (1.5 osmol/L, Figure 3C), spores transitioned to dark phase and became susceptible to killing by heat. However, in germination solution with an equal osmolarity of sucrose (1.5 osmol/L, Figure 3D), spores transitioned to dark phase but did not become susceptible to killing by heat (DPA released, >100-fold increase in RFU). In the presence of sucrose, dark phase spores were not inhibited by nisin. Sucrose did not osmotically activate spores; osmotic dehydration of the spore core protected the spores from heat killing as previously demonstrated [35].

### Evidence for Membrane Integrity as Mechanism of Inhibition by Nisin and Osmotic Activation

**Figure 4:**
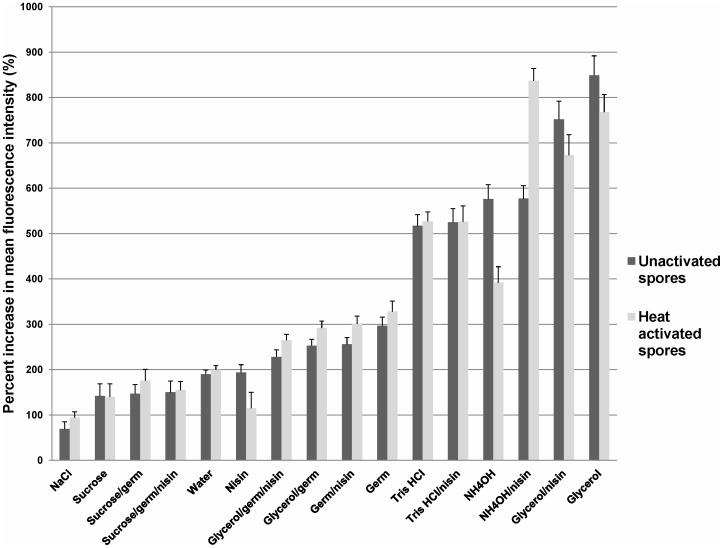
Evidence for membrane integrity as mechanism of inhibition by nisin and osmotic activation. The percent increase in the uptake of propidium iodide (PI) in *C. difficile* spores was assessed by flow cytometry. The mean fluorescence intensity was determined for each sample and compared to a baseline that did not contain PI. Loss of membrane integrity is inferred by increases in the mean fluorescence intensity as PI passes into the spore. The means of the data from experiments conducted in triplicate are presented. Error bars indicate standard error.

Outgrowth inhibition of germinated *Bacillus* spores in the presence of nisin has been shown to be accompanied by the loss of membrane integrity [20]. To determine whether *C. difficile* spores were similarly affected by the presence of osmotic activation solutes and nisin, spores were evaluated for increases in membrane permeability by monitoring uptake of PI by flow cytometry. Loss of membrane integrity is inferred by increases in the mean fluorescence intensity as PI passes into the spore. Figure 4 shows the percent increase in the uptake of PI in *C. difficile* spores. Sodium chloride and sucrose, solutes that dehydrate the spore core and do not readily permeate plasma membranes showed the lowest levels of PI associated fluorescence (<200% increase compared to baseline). Membrane permeability was significantly reduced in germination solution containing sucrose (with or without nisin) as demonstrated by lower levels of PI uptake compared to germination solution with/without nisin and/or glycerol (P<0.001). Germination solutions with glycerol, demonstrated equivalent PI uptake to germination solution alone with or without nisin (P>0.4, glycerol containing germination solutions compared to germination solutions alone), therefore, membrane permeability was not increased with the addition of glycerol to germination solution. Only osmotic activation solutes (glycerol, Tris HCl and ammonium) with or without nisin, significantly increased PI associated fluorescence in spores compared with all other solutions assessed (>500% increase compared to baseline, P<0.001). Osmotic activation solutes significantly increased membrane permeability in spores in the absence of nisin, implying that the synergistic effects of these solutes and nisin may be due to osmotically-induced loss of membrane integrity.

## Discussion

We found that a comprehensive germination solution (Table 1) induced similar germination responses in the 13 diverse spore strains assessed (∼1 log_10_CFU of spores germinated). The addition of a heat activation step resulted in germination of >2.5 log_10_CFU of spores, consistent with previous studies demonstrating that heat activation stimulates germination in a portion of the superdormant fraction of spores. Nisin did not stimulate germination of spores or kill dormant spores, but it did inhibit outgrowth of germinated spores. Contrary to our hypothesis, solutes that readily permeate plasma membranes (osmotic activators) did not stimulate germination of unactivated spores alone or in combination with a comprehensive germination solution. Unexpectedly however, osmotic activators alone in conjunction with nisin significantly increased outgrowth inhibition (>100 fold increase). Flow cytometry studies suggested that the ability of nisin to prevent outgrowth in spores exposed to osmotic activators may be linked to changes in membrane integrity induced by increased membrane permeability. These findings demonstrate that osmotic activation effectively circumvents the limitations of the “germinate to exterminate” strategy and improves the inhibitory effects of nisin by safe and practical means. Therefore, the synergistic effects of osmotic activation and nisin might provide a novel strategy to reduce the burden of *C. difficile* spores on skin and environmental surfaces.

The process of germination consists of a cascade of events that cause spores to become more susceptible to killing by stressors than in their dormant state. Currently, the success of germination as a spore control strategy has been tempered by the efficacy of triggers to stimulate germination in entire spore populations. In response to this limitation, Gould and Preston et. al. previously demonstrated that germinant mixtures containing solutes such as ammonium hydroxide, Tris HCl and glycerol, which readily permeate plasma membranes, stimulated superdormant fractions of *Bacillus spp.* spores [34], [35]. Contradictory to these findings, ammonium hydroxide, Tris HCl and glycerol did not stimulate superdormant *C.difficile* spores when combined with a germination solution (Table 1). We can propose two explanations to elucidate our contradictory findings. First, previous experiments were performed with *Bacillus spp*. and there may be inherent differences in *C.difficile* germination that inhibits the action of osmotic solutes. It is well established that germination dynamics vary among genera [40]. *C. difficile* is particularly divergent, sharing few homologous germination pathway genes with other species [41]. Second, our findings may be attributed to the composition of our germination solution. Osmotic activation occurs when the uncharged form of a solute (i.e. NH_4_OH rather than NH_4_^+^) permeates the spore core and reacts inside [34], [35]. Components of our germination solution, which include taurocholic acid, may prevent the solute from remaining in the necessary state. Additionally, we unexpectedly demonstrated the novel finding that osmotic activation solutes alone were sufficient for outgrowth inhibition by nisin. Gut et al. previously determined that changes in the spore’s inner membrane induced by germination were required for outgrowth inhibition by nisin in *Bacillus anthracis* spores [20], [23]. We found that *C. difficile* spores exposed to osmotic activation solutes alone did not germinate, however, changes occurred which allowed nisin to interact with the spore and inhibit outgrowth. Flow cytometry analysis revealed significant increases in spore membrane permeability induced by osmotic activation solutes. Similar increases in membrane permeability were previously observed by flow cytometry in *Bacillus anthracis* spores in the presence of a rich germination medium and nisin [20]. Preliminary data suggests that this novel phenomenon is not exclusive to *C. difficile* spores, but may be species specific. *Bacillus atrophaeus* spores, a common *Bacillus anthracis* surrogate, are similarly inhibited by osmotic activators and nisin, however *Bacillus subtilis* and *Bacillus thuringiensis* are not (authors’ unpublished data).

Our findings have important practical implications. First, because osmotic activation solutes are mild and nisin is GRAS, they may be safely applied to patients’ skin. We have previously demonstrated that skin contamination is prevalent during treatment of *C.difficile* infection and persists after treatment has ended [42]. Currently, there are no methods established to significantly reduce the burden of spores on skin. Routine bathing practices have been shown to have limited efficacy in decreasing the burden of spores on skin [43]. The addition of nisin and osmotic activation solutes to bathing practices may be a promising method to safely reduce *C.difficile* skin contamination. Second, elucidation of the changes that occur in the spore’s membrane by osmotic activation may lead to new strategies for eradicating spores. It has yet to be determined whether spore membrane changes induced by osmotic activation could enhance reduction of spores by other stressors or compounds. Last, because osmotic activators and nisin were effective for inhibiting outgrowth in *Bacillus atrophaeus* spores, this method may be similarly efficacious in preventing outgrowth of *Bacillus anthracis* spores.

Our study has some limitations. First, all experiments were performed *in vitro*; additional studies are required to assess the efficacy of osmotic activation and nisin to inhibit spores on skin and environmental surfaces. Real-world soil or organic load could potentially inhibit osmotic activation. Therefore, the addition of unknown constituents on surfaces would have to be considered when designing formulations to maintain the proper osmolarity for use on skin and the environment. Second, despite significant increases in spore inhibition by osmotic activation and nisin, a small fraction of spores remained resistant. However, the inoculum (10^6^ CFU) utilized in this study was considerably higher than what is naturally found on contaminated surfaces. It is our experience that skin and environmental surfaces typically yield less than 10^3^ CFU of *C. difficile* (authors’ unpublished data). In addition, the nisin concentration in our study design was calibrated to result in sub-inhibitory levels that would not influence interpretation of the enumeration assays due to inhibition of vegetative *C. difficile.* It is unknown whether increased concentrations of nisin would enhance inhibition and prevent outgrowth in entire populations of spores. Finally, further studies are necessary to determine the specific changes that occur during osmotic activation that facilitate synergistic interaction with nisin. Previously, it has been demonstrated that nisin utilizes lipid II as the target in the germinated spore’s inner membrane during outgrowth inhibition [23]. Future studies are needed to determine if nisin similarly targets lipid II in osmotically activated *C.difficile* spores.
